# Supplementary material for: A kinetic model for USP14 regulated substrate degradation in 26S proteasome
Source: PLoS Comput Biol. 2025 May 2;21(5):e1012761. doi: 10.1371/journal.pcbi.1012761 (PMC12068737; doi:10.1371/journal.pcbi.1012761)
Supplement: S1 Text — (DOCX) [file pcbi.1012761.s001.docx]

**Supporting information**

**Text A. Conformational differences in E_A_-like, E_D_-like, and S_D_-like proteasomal states.**

In E_A_-like conformations, the UBL domain of USP14 tightly binds to the proteasome, while its USP domain has only a weaker interaction with the proteasome. Additionally, substrates are not yet engaged in these conformations, either without substrate ($E_{A1}^{UBL}$) or with substrate loosely bound to the proteasome via ubiquitin chains without insertion of its N- or C-terminus into the ATPase motor ($E_{A2.0}^{UBL}$, $E_{A2.1}^{UBL}$). In the E_D_-like conformations, both the UBL and USP domains in USP14 bind to the proteasome, and the substrate ubiquitin chains are also bound to the USP domain. The E_D_-like conformations are engaged with the substrate being gripped into the OB-ring and ATPase motor, and implicated in substrate unfolding, translocation, and hydrolysis. The conformations inside the E_D_-like category differ primarily in their spatial arrangements of the regulatory particle ATPase (RPT) subunits comprising the ATPase motor, as indicated by different subscripts in these conformations. The S_D_-like conformations are not engaged with substrate and are associated with degradation inhibition mediated by ubiquitin-bound USP14. In this case, the USP domain of USP14 remains tightly bound to the proteasome, with ubiquitin chains contact on the USP domain. Moreover, RPN11 blocks the entry to the OB-ring, hindering subsequent substrate insertion and translocation processes. The S_D_-like conformations slightly differ from each other in the states of ATPase motor and the CP gate (the part of CP controlling substrate entrance), and the state of ATPase-CP interface. It is highly likely that transitions occur among the conformations within E_A_-like, E_D_-like, and S_D_-like categories. The exact transitions within the three categories of conformations are still unclear due to the complexity and subtlety of proteasomal machinery.

**Table A. The parameters in Eqs. A for the full reaction model in Fig. 1C.**

| **Parameter** | **Corresponding process** | **Value** | **Upper bound value / No upper bound** |
| --- | --- | --- | --- |
| $k_{su2}^{+}$ | $S_{U2}$ binding on $PD$ | $8.500\times{10}^{3} M^{-1}s^{-1}$ | $8.500\times{10}^{5} M^{-1}s^{-1}$ |
| $k_{su2}^{-}$ | $S_{U2}$ unbinding from $PDS_{U2}$ | $1.785\times{10}^{-1} s^{-1}$ | $1.785\times{10}^{-1} s^{-1}$ |
| $k_{su}^{+}$ | $S_{U}$ binding on $PD$ | $5.950\times{10}^{2} M^{-1}s^{-1}$ | $8.500\times{10}^{5} M^{-1}s^{-1}$ |
| $k_{su}^{-}$ | $S_{U}$ unbinding from $PDS_{U}$ | $1.785\times{10}^{-1} s^{-1}$ | $1.785\times{10}^{-1} s^{-1}$ |
| $k_{i}^{+}$ | Tail insertion | $1.250\times{10}^{-1} s^{-1}$ | $6.250\times{10}^{-1} s^{-1}$ |
| $k_{i}^{-}$ | Reverse process of tail insertion | $1.000\times{10}^{-3} s^{-1}$ | No upper bound |
| $k_{d}^{+}$ | Deubiquitination by USP14 | $6.364\times{10}^{-2} s^{-1}$ | No upper bound |
| $k_{d}^{-}$ | Reverse process of deubiquitination by USP14 | $1.000\times{10}^{-3} s^{-1}$ | No upper bound |
| $k_{h}^{+}$ | Substrate translocation and hydrolysis | $2.968\times{10}^{-2} s^{-1}$ | $4.225\times{10}^{-2} s^{-1}$ |
| $k_{h}^{-}$ | Reverse process of substrate translocation and hydrolysis | $1.000\times{10}^{-3} s^{-1}$ | No upper bound |
| $k_{s}^{+}$ | $S_{U}$ binding on $PD_{U}$ | $1.000\times{10}^{-3} s^{-1}$ | No upper bound |
| $k_{s}^{-}$ | $S_{U}$ unbinding from $PD_{U}S_{U}$ | $8.925\times{10}^{-2} s^{-1}$ | $1.785\times{10}^{-1} s^{-1}$ |
| $k_{u}^{+}$ | Ubiquitin chain binding | $1.000\times{10}^{-3} s^{-1}$ | No upper bound |
| $k_{u}^{-}$ | Ubiquitin chain unbinding | $5.625\times{10}^{-1} s^{-1}$ | No upper bound |
| $k_{t}^{+}$ | Transition from $PD$ to $PD_{t}$ | $5.400\times{10}^{-1} s^{-1}$ | No upper bound |
| $k_{t}^{-}$ | Transition from $PD_{t}$ to $PD$ | $10.125 s^{-1}$ | No upper bound |

**Table B. The parameters in Eqs. B for the simplified model for USP14-regulated proteasomal degradation demonstrated in Fig. 4A.**

| **Parameter** | **Corresponding process** | **Value** |
| --- | --- | --- |
| $k_{su2}^{+}$ | $S_{U2}$ binding on $PD$ | Same as Tab. A |
| $k_{su2}^{-}$ | $S_{U2}$ unbinding from $PDS_{U2}$ | Same as Tab. A |
| $k_{i}^{+}$ | Tail insertion | Same as Tab. A |
| $k_{i}^{-}$ | Reverse process of tail insertion | Same as Tab. A |
| $k_{d}^{+}$ | Deubiquitination by USP14 | Same as Tab. A |
| $k_{d}^{-}$ | Reverse process of deubiquitination by USP14 | Same as Tab. A |
| $k_{h}^{+}$ | Substrate translocation and hydrolysis | Same as Tab. A |
| $k_{h}^{-}$ | Reverse process of substrate translocation and hydrolysis | Same as Tab. A |
| $k_{s}^{+}$ | $S_{U}$ binding on $PD_{U}$ | Same as Tab. A |
| $k_{s}^{-}$ | $S_{U}$ unbinding from $PD_{U}S_{U}$ | Same as Tab. A |
| $k_{u}^{+}$ | Ubiquitin chain binding | Same as Tab. A |
| $k_{u}^{-}$ | Ubiquitin chain unbinding | Same as Tab. A |
| $k_{t}^{+}$ | Transition from $PD$ to $PD_{t}$ | Same as Tab. A |
| $k_{t}^{-}$ | Transition from $PD_{t}$ to $PD$ | Same as Tab. A |

**Table C. The parameters in Eqs. C for the reactions of substrate degradation without USP14 in Fig. 4B.**

| **Parameter** | **Corresponding process** | **Value** |
| --- | --- | --- |
| $l_{su2}^{+}$ | $S_{U2}$ binding | Same as $k_{su2}^{+}$ in Tab. A |
| $l_{su2}^{-}$ | $S_{U2}$ unbinding | Same as $k_{su2}^{-}$ in Tab. A |
| $l_{i}^{+}$ | Tail insertion | Same as $k_{i}^{+}$ in Tab. A |
| $l_{i}^{-}$ | Reverse process of tail insertion | Same as $k_{i}^{-}$ in Tab. A |
| $l_{d}^{+}$ | Deubiquitination by RPN11 | $0.909 s^{-1}$ |
| $l_{d}^{-}$ | Reverse process of deubiquitination by RPN11 | Same as $k_{d}^{-}$ in Tab. A |
| $l_{h}^{+}$ | Substrate translocation and hydrolysis | Same as $k_{h}^{+}$ in Tab. A |
| $l_{h}^{-}$ | Reverse process of substrate translocation and hydrolysis | Same as $k_{h}^{-}$ in Tab. A |

**Equations A. ODEs for the simplified kinetic model in Fig. 1C.**

$$\begin{aligned} \frac{d\left[ PD \right]}{dt}=k_{su}^{-}\left[ PDS_{U} \right]-k_{su}^{+}\left[ S_{U} \right]\left[ PD \right]+k_{su2}^{-}\left[ PDS_{U2} \right]-k_{su2}^{+}\left[ S_{U2} \right]\left[ PD \right] \\ +k_{u}^{-}\left[ PD_{U} \right]-k_{u}^{+}\left[ U \right]\left[ PD \right]+k_{t}^{-}\left[ PD_{t} \right]-k_{t}^{+}\left[ PD \right],\#\left( S1a \right) \end{aligned}$$

$$\begin{aligned} \frac{d\left[ PDS_{U2} \right]}{dt}=k_{su2}^{+}\left[ S_{U2} \right]\left[ PD \right]-k_{su2}^{-}\left[ PDS_{U2} \right]+k_{i}^{-}\left[ PD_{U}S_{U}^{i} \right]-k_{i}^{+}\left[ PDS_{U2} \right] \\ +k_{d}^{-}\left[ PD_{U}S_{U} \right]-k_{d}^{+}\left[ PDS_{U2} \right],\#\left( S1b \right) \end{aligned}$$

$$\begin{aligned} \frac{d\left[ PDS_{U} \right]}{dt}=\text{ }k_{su}^{+}\left[ S_{U} \right]\left[ PD \right]-k_{su}^{-}\left[ PDS_{U} \right]+k_{i}^{-}\left[ PD_{U}S^{i} \right]-k_{i}^{+}\left[ PDS_{U} \right],\#\left( S1c \right) \end{aligned}$$

$$\begin{aligned} \frac{d\left[ PD_{U}S_{U}^{i} \right]}{dt}=k_{i}^{+}\left[ PDS_{U2} \right]-k_{i}^{-}\left[ PD_{U}S_{U}^{i} \right]+k_{h}^{-}\left[ PD_{U} \right]-k_{h}^{+}\left[ PD_{U}S_{U}^{i} \right],\#\left( S1d \right) \end{aligned}$$

$$\begin{aligned} \frac{d\left[ PD_{U}S^{i} \right]}{dt}=k_{i}^{+}\left[ PDS_{U} \right]-k_{i}^{-}\left[ PD_{U}S^{i} \right]+k_{h}^{-}\left[ PD_{U} \right]-k_{h}^{+}\left[ PD_{U}S^{i} \right],\#\left( S1e \right) \end{aligned}$$

$$\begin{aligned} \frac{d\left[ PD_{U} \right]}{dt}=k_{h}^{+}\left[ PD_{U}S_{U}^{i} \right]-k_{h}^{-}\left[ PD_{U} \right]+k_{h}^{+}\left[ PD_{U}S^{i} \right]-k_{h}^{-}\left[ PD_{U} \right]+k_{s}^{-}\left[ PD_{U}S_{U} \right] \\ -k_{s}^{+}\left[ S_{U} \right]\left[ PD_{U} \right]+k_{u}^{+}\left[ U \right]\left[ PD \right]-k_{u}^{-}\left[ PD_{U} \right],\#\left( S1f \right) \end{aligned}$$

$$\begin{aligned} \frac{d\left[ PD_{U}S_{U} \right]}{dt}=k_{d}^{+}\left[ PDS_{U2} \right]-k_{d}^{-}\left[ PD_{U}S_{U} \right]+k_{s}^{+}\left[ S_{U} \right]\left[ PD_{U} \right]-k_{s}^{-}\left[ PD_{U}S_{U} \right],\#\left( S1g \right) \end{aligned}$$

$$\begin{aligned} \frac{d\left[ PD_{t} \right]}{dt}=k_{t}^{+}\left[ PD \right]-k_{t}^{-}\left[ PD_{t} \right],\#\left( S1h \right) \end{aligned}$$

$$\begin{aligned} \frac{d\left[ S_{U2} \right]}{dt}=k_{su2}^{-}\left[ PDS_{U2} \right]-k_{su2}^{+}\left[ S_{U2} \right]\left[ PD \right],\#\left( S1i \right) \end{aligned}$$

$$\begin{aligned} \frac{d\left[ S_{U} \right]}{dt}=k_{su}^{-}\left[ PDS_{U} \right]-k_{su}^{+}\left[ S_{U} \right]\left[ PD \right]+k_{s}^{-}\left[ PD_{U}S_{U} \right]-k_{s}^{+}\left[ S_{U} \right]\left[ PD_{U} \right],\#\left( S1j \right) \end{aligned}$$

$$\begin{aligned} \frac{d\left[ U \right]}{dt}=k_{u}^{-}\left[ PD_{U} \right]-k_{u}^{+}\left[ U \right]\left[ PD \right],\#\left( S1k \right) \end{aligned}$$

where [.] denotes the concentration of a state variable, and *k*’s are the rate constants. Superscript signs $+$ or $-$ in *k*’s are for forward and reverse rate constants within a reversible reaction. $k_{su2}^{+}$ ($k_{su}^{+}$) and $k_{su2}^{-}$ ($k_{su}^{-}$) are the rate constants for $S_{U2}$ ($S_{U}$) binding and unbinding on E_A_-like conformations, while $k_{s}^{+}$ and $k_{s}^{-}$ are the rate constants for $S_{U}$ binding and unbinding on S_D_-like conformations. $k_{u}^{+}$ and $k_{u}^{-}$ are the rate constants for ubiquitin chain binding and unbinding. Similarly, subscripts $i, d, h, t$ correspond to the reactions of substrate insertion, USP14 deubiquitination, substrate hydrolysis, and the transition between $PD$ and $PD_{t}$, respectively. The detailed reactions and rate constants are listed in Tab. A.

**Equations B. ODEs for the simplified kinetic model in Fig. 4A.**

$$\frac{d\left[ PD \right]}{dt}=k_{su2}^{-}\left[ PDS_{U2} \right]-k_{su2}^{+}\left[ S_{U2} \right]\left[ PD \right]+k_{u}^{-}\left[ PD_{U} \right]-k_{u}^{+}\left[ U \right]\left[ PD \right]+k_{t}^{-}\left[ PD_{t} \right]-k_{t}^{+}\left[ PD \right], (S2a)$$

$$\frac{d\left[ PDS_{U2} \right]}{dt}=k_{su2}^{+}\left[ S_{U2} \right]\left[ PD \right]-k_{su2}^{-}\left[ PDS_{U2} \right]+k_{i}^{-}\left[ PD_{U}S_{U}^{i} \right]-k_{i}^{+}\left[ PDS_{U2} \right]+k_{d}^{-}\left[ PD_{U}S_{U} \right]-k_{d}^{+}\left[ PDS_{U2} \right], (S2b)$$

$$\frac{d\left[ PD_{U}S_{U}^{i} \right]}{dt}=k_{i}^{+}\left[ PDS_{U2} \right]-k_{i}^{-}\left[ PD_{U}S_{U}^{i} \right]+k_{h}^{-}\left[ PD_{U} \right]-k_{h}^{+}\left[ PD_{U}S_{U}^{i} \right], (S2c)$$

$$\frac{d\left[ PD_{U} \right]}{dt}=k_{h}^{+}\left[ PD_{U}S_{U}^{i} \right]-k_{h}^{-}\left[ PD_{U} \right]+k_{s}^{-}\left[ PD_{U}S_{U} \right]-k_{s}^{+}\left[ S_{U} \right]\left[ PD_{U} \right]+k_{u}^{+}\left[ U \right]\left[ PD \right]-k_{u}^{-}\left[ PD_{U} \right], (S2d)$$

$$\frac{d\left[ PD_{U}S_{U} \right]}{dt}=k_{d}^{+}\left[ PDS_{U2} \right]-k_{d}^{-}\left[ PD_{U}S_{U} \right]+k_{s}^{+}\left[ S_{U1} \right]\left[ PD_{U} \right]-k_{s}^{-}\left[ PD_{U}S_{U} \right], (S2e)$$

$$\frac{d\left[ PD_{t} \right]}{dt}=k_{t}^{+}\left[ PD \right]-k_{t}^{-}\left[ PD_{t} \right]. (S2f)$$

**Equations C. ODEs for the reactions of substrate degradation without USP14 as shown in Fig. 4B.**

$$\frac{d\left[ P \right]}{dt}=l_{h}^{+}\left[ PS_{U}^{i} \right]-l_{h}^{-}\left[ P \right]+l_{su2}^{-}\left[ PS_{U2} \right]-l_{su2}^{+}\left[ S_{U2} \right]\left[ P \right], (S3a)$$

$$\frac{d\left[ PS_{U2} \right]}{dt}=l_{su2}^{+}\left[ S_{U2} \right]\left[ P \right]-l_{su2}^{-}\left[ PS_{U2} \right]+l_{i}^{-}\left[ PS_{U2}^{i} \right]-l_{i}^{+}\left[ PS_{U2} \right], (S3b)$$

$$\frac{d\left[ PS_{U2}^{i} \right]}{dt}=l_{i}^{+}\left[ PS_{U2} \right]-l_{i}^{-}\left[ PS_{U2}^{i} \right]+l_{d}^{-}\left[ U \right]\left[ PS_{U}^{i} \right]-l_{d}^{+}\left[ PS_{U2}^{i} \right], (S3c)$$

$$\frac{d\left[ PS_{U}^{i} \right]}{dt}=l_{d}^{+}\left[ PS_{U2}^{i} \right]-l_{d}^{-}\left[ U \right]\left[ PS_{U}^{i} \right]+l_{h}^{-}\left[ P \right]-l_{h}^{+}\left[ PS_{U}^{i} \right]. (S3d)$$

where the rate constants are denoted by $l$ to distinguish from the rate constants of the USP14-bound proteasome that are denoted by *k*. Note that $l_{d}$denotes the deubiquitination rate of RPN11, which differs from the deubiquitination rate indicated by $k_{d}$ for USP14.

**Equations D. ODES for USP14-bound and USP14-free proteasome with two substrates *S* and *T.***

$$\frac{d\left[ PD \right]}{dt}=k_{su2}^{-}\left[ PDS_{U2} \right]-k_{su2}^{+}\left[ S_{U2} \right]\left[ PD \right]+k_{tu2}^{-}\left[ PDT_{U2} \right]-k_{tu2}^{+}\left[ T_{U2} \right]\left[ PD \right]+k_{u}^{-}\left[ PD_{U} \right]-k_{u}^{+}\left[ U \right]\left[ PD \right]+k_{t}^{-}\left[ PD_{t} \right]-k_{t}^{+}\left[ PD \right], (S4a)$$

$$\frac{d\left[ PDS_{U2} \right]}{dt}=k_{su2}^{+}\left[ S_{U2} \right]\left[ PD \right]-k_{su2}^{-}\left[ PDS_{U2} \right]+k_{i,s}^{-}\left[ PD_{U}S_{U}^{i} \right]-k_{i,s}^{+}\left[ PDS_{U2} \right]+k_{d,s}^{-}\left[ PD_{U}S_{U} \right]-k_{d,s}^{+}\left[ PDS_{U2} \right], (S4b)$$

$$\frac{d\left[ PDT_{U2} \right]}{dt}=k_{tu2}^{+}\left[ T_{U2} \right]\left[ PD \right]-k_{tu2}^{-}\left[ PDT_{U2} \right]+k_{i,t}^{-}\left[ PD_{U}T_{U}^{i} \right]-k_{i,t}^{+}\left[ PDT_{U2} \right]+k_{d,t}^{-}\left[ PD_{U}T_{U} \right]-k_{d,t}^{+}\left[ PDT_{U2} \right], (S4c)$$

$$\frac{d\left[ PD_{U}S_{U}^{i} \right]}{dt}=k_{i,s}^{+}\left[ PDS_{U2} \right]-k_{i,s}^{-}\left[ PD_{U}S_{U}^{i} \right]+k_{h,s}^{-}\left[ PD_{U} \right]-k_{h,s}^{+}\left[ PD_{U}S_{U}^{i} \right], (S4d)$$

$$\frac{d\left[ PD_{U}T_{U}^{i} \right]}{dt}=k_{i,t}^{+}\left[ PDT_{U2} \right]-k_{i,t}^{-}\left[ PD_{U}T_{U}^{i} \right]+k_{h,t}^{-}\left[ PD_{U} \right]-k_{h,t}^{+}\left[ PD_{U}T_{U}^{i} \right], (S4e)$$

$$\frac{d\left[ PD_{U} \right]}{dt}=k_{h,s}^{+}\left[ PD_{U}S_{U}^{i} \right]-k_{h,s}^{-}\left[ PD_{U} \right]+k_{h,t}^{+}\left[ PD_{U}S_{U}^{i} \right]-k_{h,t}^{-}\left[ PD_{U} \right]+k_{s}^{-}\left[ PD_{U}S_{U} \right]-k_{s}^{+}\left[ S_{U} \right]\left[ PD_{U} \right]+k_{t}^{-}\left[ PD_{U}T_{U} \right]-k_{t}^{+}\left[ T_{U} \right]\left[ PD_{U} \right]+k_{u}^{+}\left[ U \right]\left[ PD \right]-k_{u}^{-}\left[ PD_{U} \right], (S4f)$$

$$\frac{d\left[ PD_{U}S_{U} \right]}{dt}=k_{d,s}^{+}\left[ PDS_{U2} \right]-k_{d,s}^{-}\left[ PD_{U}S_{U} \right]+k_{s}^{+}\left[ S_{U1} \right]\left[ PD_{U} \right]-k_{s}^{-}\left[ PD_{U}S_{U} \right], (S4g)$$

$$\frac{d\left[ PD_{U}T_{U} \right]}{dt}=k_{d,t}^{+}\left[ PDT_{U2} \right]-k_{d,t}^{-}\left[ PD_{U}T_{U} \right]+k_{t}^{+}\left[ T_{U1} \right]\left[ PD_{U} \right]-k_{t}^{-}\left[ PD_{U}T_{U} \right], (S4h)$$

$$\frac{d\left[ PD_{t} \right]}{dt}=k_{t}^{+}\left[ PD \right]-k_{t}^{-}\left[ PD_{t} \right]. (S4i)$$

**Equations E. ODES for USP14-free proteasome and in the presence of substrates *S* and *T.***

$$\frac{d\left[ P \right]}{dt}=l_{h,s}^{+}\left[ PS_{U}^{i} \right]-l_{h,s}^{-}\left[ P \right]+l_{h,t}^{+}\left[ PT_{U}^{i} \right]-l_{h,t}^{-}\left[ P \right]+l_{su2}^{-}\left[ PS_{U2} \right]-l_{su2}^{+}\left[ S_{U2} \right]\left[ P \right]+l_{tu2}^{-}\left[ PT_{U2} \right]-l_{tu2}^{+}\left[ T_{U2} \right]\left[ P \right], (S5a)$$

$$\frac{d\left[ PS_{U2} \right]}{dt}=l_{su2}^{+}\left[ S_{U2} \right]\left[ P \right]-l_{su2}^{-}\left[ PS_{U2} \right]+l_{i,s}^{-}\left[ PS_{U2}^{i} \right]-l_{i,s}^{+}\left[ PS_{U2} \right], (S5b)$$

$$\frac{d\left[ PT_{U2} \right]}{dt}=l_{su2}^{+}\left[ S_{U2} \right]\left[ P \right]-l_{su2}^{-}\left[ PS_{U2} \right]+l_{i,t}^{-}\left[ PT_{U2}^{i} \right]-l_{i,t}^{+}\left[ PT_{U2} \right], (S5c)$$

$$\frac{d\left[ PS_{U2}^{i} \right]}{dt}=l_{i,s}^{+}\left[ PS_{U2} \right]-l_{i,s}^{-}\left[ PS_{U2}^{i} \right]+l_{d,s}^{-}\left[ U \right]\left[ PS_{U}^{i} \right]-l_{d,s}^{+}\left[ PS_{U2}^{i} \right], (S5d)$$

$$\frac{d\left[ PT_{U2}^{i} \right]}{dt}=l_{i,t}^{+}\left[ PT_{U2} \right]-l_{i,t}^{-}\left[ PT_{U2}^{i} \right]+l_{d,t}^{-}\left[ U \right]\left[ PT_{U}^{i} \right]-l_{d,t}^{+}\left[ PT_{U2}^{i} \right], (S5e)$$

$$\frac{d\left[ PS_{U}^{i} \right]}{dt}=l_{d,s}^{+}\left[ PS_{U2}^{i} \right]-l_{d,s}^{-}\left[ U \right]\left[ PS_{U}^{i} \right]+l_{h,s}^{-}\left[ P \right]-l_{h,s}^{+}\left[ PS_{U}^{i} \right], (S5f)$$

$$\frac{d\left[ PT_{U}^{i} \right]}{dt}=l_{d,t}^{+}\left[ PT_{U2}^{i} \right]-l_{d,t}^{-}\left[ U \right]\left[ PT_{U}^{i} \right]+l_{h,t}^{-}\left[ P \right]-l_{h,t}^{+}\left[ PT_{U}^{i} \right]. (S5g)$$

For both Eqs. D and Eqs. E, subscript *s* and *t* is used to distinguish the corresponding reactions for substrates *S* and *T*, respectively.

**Figure A. Parameter sensitivity analysis for the dynamics of USP14-bound proteasome described by Eqs. S1.**

**
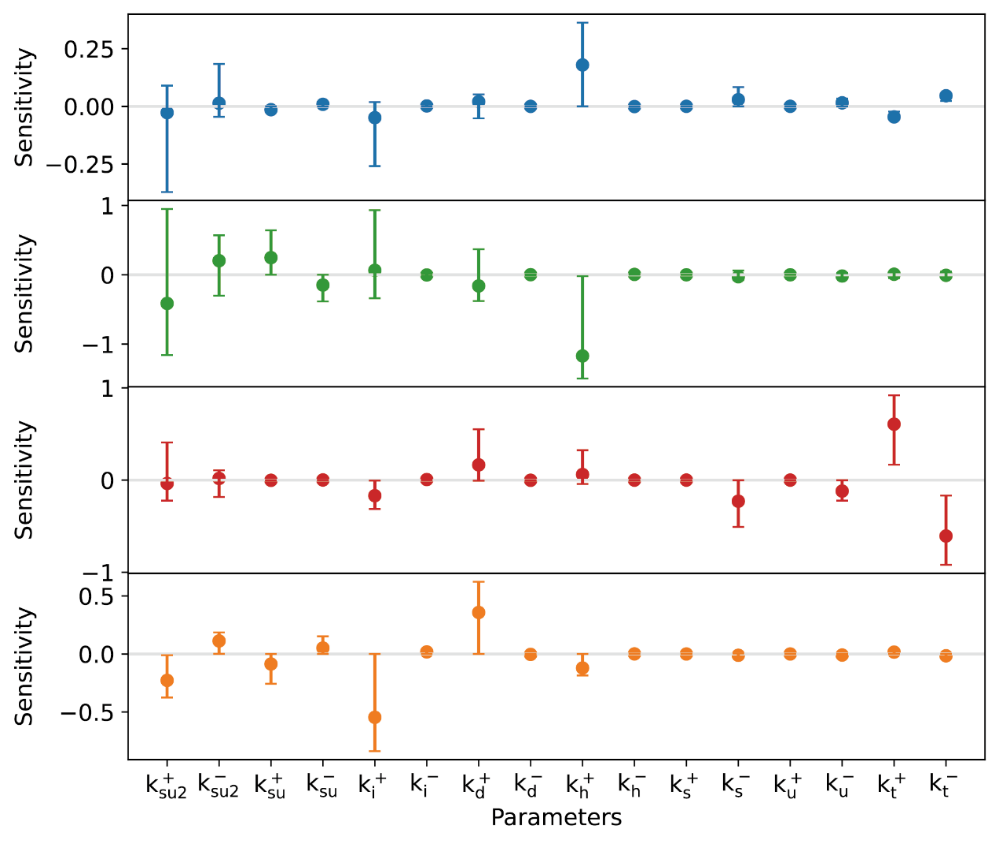
**

**Fig S1. Parameter Sensitivity Analysis of the kinetic model (Eqs. S1) for USP14-bound Proteasome.** The sensitivity is defined as $s(t;p)\equiv{\partial lnX\left( t;p \right)}/{\partial lnp}$. From top to bottom: the sensitivities of E_A_-like, E_D_-like, and S_D_-like conformations, as well as the sensitivity of the residual substrate concentration ratio to perturbations in various parameters. The points represent the average sensitivity, with error bars indicating the range of sensitivities. The most sensitive parameters are the substrate binding and unbinding rates $k_{su2}^{+}$ and $k_{su2}^{-}$, the tail insertion rate $k_{i}^{+}$, the USP14 deubiquitination rate $k_{d}^{+}$, and the substrate hydrolysis rate $k_{h}^{+}$.
